# RETRACTION: Construction and Verification of a Novel Pyroptosis‐Related lncRNA Signature Associated with Immune Landscape in Gliomas

**DOI:** 10.1155/jo/9842864

**Published:** 2026-08-26

**Authors:** Journal of Oncology

RETRACTION: X. Feng, Y. Chen, X. Liu, Z. Zhong, and Y. Liu, “Construction and Verification of a Novel Pyroptosis‐Related lncRNA Signature Associated with Immune Landscape in Gliomas,” *Journal of Oncology*, vol. 2022 (2022). https://doi.org/10.1155/2022/7043431.

The above article, published online on 14 October 2022 in Wiley Online Library (https://wileyonlinelibrary.com), has been retracted following the agreement of the authors and John Wiley & Sons Ltd.

The retraction has been agreed following communication received from the authors indicating that one of the authors had used third‐party assistance in code debugging without informing the other authors. Specifically, the drug sensitivity figures in Figures 8 and 9 were obtained after a third party adjusted the code but were not generated by the third party. As a consequence, the authors no longer accept the results presented in the article due to the involvement of a third party, and it is therefore retracted.

All authors agree with this retraction.

